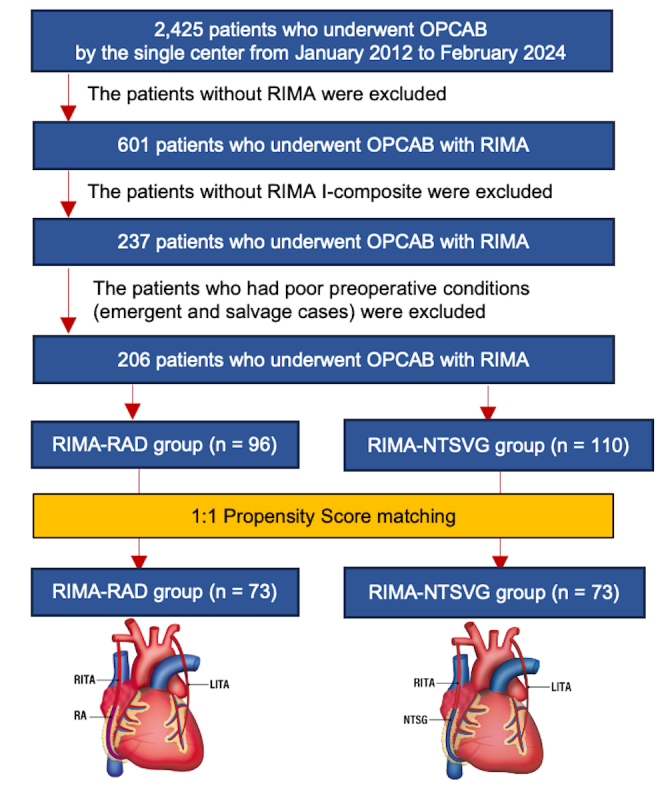# No-Touch Saphenous Vein Graft vs Radial Artery as an I-Composite Graft From the Right Internal Mammary Artery

**DOI:** 10.1016/j.atssr.2025.08.021

**Published:** 2025-09-13

**Authors:** Ryohei Ushioda, Hideki Isa, Hiroyuki Kamiya, Dit Yoongtong, Boonsap Sakboon, Jaroen Cheewinmethasiri, Nuttapon Arayawudhikul

**Affiliations:** 1 Cardiovascular and Thoracic Surgery Unit, Department of Surgery, Lampang Hospital, Lampang, Thailand; 2 Department of Cardiac Surgery, Asahikawa Medical University, Asahikawa, Hokkaido, Japan

## Abstract

**Background:**

As an inflow source for an I-composite graft, the right internal mammary artery (RIMA) can be a valuable option for anaortic off-pump coronary artery bypass grafting (OPCAB); however, the optimal extension graft from the in situ RIMA remains unclear. This study compared the suitability of the radial artery and no-touch saphenous vein graft as the extension graft from in situ RIMA.

**Methods:**

From January 2012 to February 2024, we performed isolated OPCAB with an I-composite RIMA graft on 208 patients at a single center. These patients were divided into the I-composite RIMA–radial (RAD group, 96 patients) and I-composite RIMA no-touch saphenous vein graft (NTSVG group, 110 patients) groups. The RAD and NTSVG groups were propensity score-matched (1:1, 73 patients each).

**Results:**

The RAD and NTSVG groups had 64 (66.7%) and 77 (70.0%) men (mean age, 62.7 [SD, 8.6] years and 65.0 [SD, 7.8] years) in the unmatched cohort and 51 (69.9%) and 50 (68.5%) men (mean age, 63.7 [SD, 8.2] years and 64.2 [SD, 7.8] years) in the matched cohort, respectively. After matching, Kaplan-Meier curves showed no significant difference in major adverse cardiac or cerebrovascular events (*P* = .62) and survival rate (*P* = .77) between the 2 groups. The midterm patency rate of the I-composite RIMA graft after matching was significantly better in the RAD group than in the NTSVG group (*P* = .015).

**Conclusions:**

The I-composite RIMA–RAD graft is an effective option for anaortic OPCAB in patients requiring multiple coronary revascularizations.

## Visual Abstract

**Figure:**
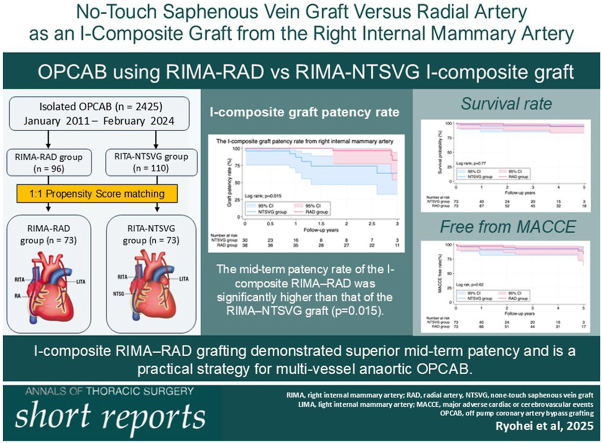

**In Short**

▪ Kaplan-Meier analysis revealed no significant differences in major adverse cardiac or cerebrovascular events or overall survival between the radial artery and no-touch saphenous vein graft groups.
▪ The midterm patency rate of the I-composite right internal mammary artery graft was significantly higher in the radial artery group than in the no-touch saphenous vein graft group group after matching.
▪ I-composite right internal mammary artery graft–radial artery grafting appears to be an effective strategy for achieving multivessel revascularization in anaortic off-pump coronary artery bypass grafting.

Off-pump coronary artery bypass grafting (OPCAB) without aortic manipulation, known as anaortic OPCAB, has gained attention as a strategy to reduce the risk of perioperative stroke.^1^ In this technique, composite grafts are frequently used to achieve multiple coronary revascularizations. The combination of a left internal mammary artery (LIMA) to the left anterior descending artery (LIMA–LAD) graft and an I-composite graft originating from the right IMA (RIMA) to revascularize other coronary territories represents a reasonable option for performing anaortic OPCAB.

At Lampang Hospital, anaortic off-pump coronary artery bypass grafting using LIMA–LAD and I-composite RIMA grafts has been actively performed. The radial artery (RAD) and no-touch saphenous vein graft (NTSVG) have both been commonly used as conduits for constructing composite grafts; however, which provides superior outcomes remains unclear. Thus, the present study evaluated the midterm outcomes of patients who underwent anaortic OPCAB with I-composite RIMA grafts using the RAD or NTSVG.

## Patients and Methods

Between January 2012 and February 2024, OPCAB was performed on 2425 patients at a single center. The RIMA was used in 601 patients, of whom 237 received an I-composite RIMA graft. Thirty-one patients with poor preoperative conditions (emergency or salvage cases, or The Society of Thoracic Surgeons score >20) were excluded. All patients underwent LIMA–LAD anastomosis, and the I-composite RIMA graft was used as the second conduit, targeting vessels with >90% stenosis. In cases where the I-composite graft could not reach the circumflex territory, a Y-composite LIMA with saphenous vein graft (SVG) was used instead. This study was approved by the Lampang Hospital Institutional Review Board (No. 028/68, 2025/2/7), which waived the requirement for written informed consent due to its retrospective design.

Patients were divided into 2 groups based on the conduit used in the I-composite graft: the RAD group (n = 96) and the NTSVG group (n = 110). Propensity score (PS) matching was performed at a 1:1 ratio (RAD, n = 73; NTSVG, n = 73) using 12 preoperative covariates (Supplemental Figure). The primary outcomes were midterm I-composite RIMA graft patency, 5-year mortality, and freedom from major adverse cardiac or cerebrovascular events (MACCE).

### Surgical Technique

All patients underwent median sternotomy. The RIMA was skeletonized using a Harmonic scalpel (Ethicon Endo-Surgery Inc) and used in situ. The RAD was harvested semiskeletonized using electrocautery, whereas the NTSVG was harvested above the knee using a no-touch technique. The I-composite graft was constructed by anastomosing the RAD or NTSVG side-to-side to the distal end of the in-situ RIMA using 8-0 Prolene (Ethicon) sutures. This configuration, commonly referred to as an “I-composite” graft, extends the in situ RIMA in a straight, linear fashion and enables revascularization of distal coronary targets without aortic manipulation, thereby facilitating anaortic OPCAB. Amlodipine (5 mg/d) was prescribed indefinitely to patients receiving RAD grafts for vasospasm prevention.

The average clinical follow-up was 2.7 (SD, 2.3) years (before matching) and 2.9 (SD, 2.3) years (after matching). Postoperative coronary computed tomography angiography (CTA) was performed in 68 symptomatic patients (38 RAD [52.1%] and 30 NTSVG [41.1%]). The mean CTA follow-up period was 2.1 (SD, 1.5) years.

### Statistical Analysis

Because the operative approach was determined at the surgeon’s discretion, group assignment was nonrandomized. To account for potential baseline differences, PS were calculated using logistic regression based on 12 preoperative variables: age, sex, diabetes mellitus, cerebral vascular accident, peripheral arterial disease, dialysis, 2-vessel disease, 3-vessel disease, left main trunk disease, low ejection fraction, and elective or urgent status. Patients were matched 1:1 using the nearest neighbor method without replacement and a caliper width of 0.2 of the SD of the logit of the PS. Standardized mean differences (SMD) were calculated to assess covariate balance before and after matching. Continuous variables with normal distributions were compared using the independent *t* test (unmatched) or paired *t* test (matched). Nonnormally distributed variables were analyzed using the Mann-Whitney *U* test (unmatched) or the Wilcoxon signed rank test (matched). For categorical variables, the Fisher exact test and the χ^2^ test were used in the unmatched cohort, whereas the McNemar test was used in the matched cohort. All analyses were conducted using STATA/MP 18.0 software (StataCorp).

## Results

Table 1 summarizes the preoperative characteristics of the included patients. The RAD and NTSVG groups consisted of 64 (66.7%) and 77 (70.0%) men (mean age, 62.7 [SD, 8.6] years and 65.0 [SD, 7.8] years) in the unmatched cohort and 51 (69.9%) and 50 (68.5%) men (mean age, 63.7 [SD, 8.2] years and 64.2 [SD, 7.8] years) in the matched cohort, respectively. In the unmatched cohort, the NTSVG group had a significantly worse preoperative status, assessed based on the European System for Cardiac Operative Risk Evaluation 2011 revision (EuroSCORE II), than did the RAD group (1.38 [interquartile range, 0.89-2.44] vs 1.90 [interquartile range, 1.30-3.78], *P* = .002). Moreover, the NTSVG group included more emergency cases than did the RAD group (18.8% vs 32.7%; *P* = .023).

**Table 1:** Patient Characteristics and Preoperative Data

Variables	Entire Cohort			Propensity Score-Matched Cohort		
	RAD Group (n = 96)	NTSVG Group (n = 110)	SMD	RAD Group (n = 73)	NTSVG Group (n = 73)	SMD
Age, mean (SD), y	62.7 (8.6)	65.0 (7.8)	−0.29	63.7 (8.2)	64.2 (7.8)	−0.07
Male sex, n (%)	64 (66.7)	77 (70.0)	−0.07	51 (69.9)	50 (68.5)	0.04
EuroSCORE II, median (IQR)	1.4 (0.9-2.4)	1.9 (1.3-3.8)	−0.37	1.4 (0.9-2.2)	1.7 (1.0-2.3)	−0.15
Comorbidity, n (%)						
Hypertension	92 (95.8)	106 (96.4)	−0.03	70 (95.9)	69 (94.5)	0.07
Diabetes mellitus	40 (41.7)	53 (48.2)	−0.13	34 (46.6)	33 (45.2)	0.04
Dialysis	6 (6.4)	11 (11.1)	−0.17	5 (6.9)	7 (9.6)	−0.10
Cerebral vascular accident	5 (5.21)	7 (6.36)	0.05	4 (5.5)	5 (6.9)	−0.06
Peripheral arterial disease	10 (10.4)	20 (18.2)	−0.22	7 (9.6)	6 (8.2)	0.10
Double-vessel disease	7 (7.3)	9 (8.2)	−0.03	7 (9.6)	6 (8.2)	0.04
Triple-vessel disease	89 (92.7)	98 (89.1)	0.13	66 (90.4)	67 (91.8)	−0.04
Low ejection fraction (<0.40)	64 (68.1)	58 (58.6)	0.20	46 (63.0)	43 (58.9)	0.10
Urgency, n (%)						
Elective	77 (80.2)	74 (67.3)	0.30	58 (79.5)	56 (76.7)	0.07
Urgent	19 (19.8)	36 (32.7)	−0.32	15 (20.6)	17 (23.3)	−0.07

EuroSCORE II, European System for Cardiac Operative Risk Evaluation 2011 revision; IQR, interquartile range; NTSVG, no-touch saphenous vein graft; RAD, radial artery; SMD, standard mean difference.

Table 2 summarizes the operative data and early complications. The total number of distal anastomoses was significantly higher in the NTSVG group than in the RAD group after matching (unmatched: 3.5 [SD, 0.7] vs 3.7 [SD, 0.9], *P* = .09; PS matched: 3.5 [SD, 0.7] vs 3.8 [SD, 0.9], *P* = .04). The Y-composite LIMA with SVG was also significantly higher in the NTSVG group (unmatched: 5.2% vs 22.7%, *P* < .001; PS matched: 4.1% vs 24.7%, *P* = .002). In the distal anastomosis area of the I-composite RIMA graft, the right coronary artery (RCA) was the predominant target site, with no significant difference between the groups (unmatched: 100% vs 96.4%, *P* = .13; PS matched: 100% vs 100%, *P* = 1.00). Notably, no significant differences in hospital stay, intensive care unit stay, or major postoperative complications were observed between the 2 groups in either cohort.

**Table 2:** Operative Data and Early Complications

Variables	Entire Cohort			Propensity Score-Matched Cohort		
	RAD Group (n = 96)	NTSVG Group (n = 110)	*P* Value	RAD Group (n = 73)	NTSVG Group (n = 73)	*P* Value
Operative data						
Operating time, median (IQR), min	280 (235-310)	280 (250-315)	.41	280 (239-310)	285 (260-330)	.16
Distal anastomoses, mean (SD), n	3.5 (0.7)	3.7 (0.9)	.09	3.5 (0.7)	3.8 (0.9)	.04
Y-composite graft from LIMA, n (%)	5 (5.2)	25 (22.7)	<.001	3 (4.1)	18 (24.7)	.002
Complete revascularization, n (%)	96 (100)	107 (97.3)	1.00	73 (100)	71 (97.3)	.50
The detail I-composite graft from RIMA						
Sequential technique, n (%)	27 (28.1)	27 (24.6)	.63	20 (27.4)	20 (27.4)	1.00
Distal anastomoses from RIMA, median (IQR), n	1 (1-3)	1 (1-4)	.82	1 (1-3)	1 (1-4)	1.00
Distal anastomosis area of the RIMA I-composite, n (%)						
Diagonal	0 (0)	2 (1.8)	.50	0 (0)	2 (2.7)	.50
Left circumflex artery	10 (10.4)	13 (11.8)	.75	8 (11.0)	9 (12.3)	1.00
Right coronary artery	96 (100)	106 (96.4)	.13	73 (100)	73 (100)	1.00
Postoperative data						
Intensive care unit stay, median (IQR), d	2.0 (1.0-2.5)	2.0 (1.0-2.0)	.89	2.0 (1.0-3.0)	2 (1.0-2.0)	.49
Hospital stay, median (IQR), d	5.0 (5.0-6.0)	5.0 (5.0-7.0)	.52	5.0 (5.0-7.0)	5.0 (5.0-6.0)	.49
30-day mortality, n (%)	1 (1.0)	2 (1.8)	1.00	1 (1.4)	1 (1.4)	1.00
Early-term postoperative complications, n (%)						
New stroke	2 (2.1)	1 (0.9)	.60	2 (2.7)	1 (1.4)	1.00
New dialysis	0 (0)	1 (0.9)	1.00	0 (0)	0 (0)	1.00
Wound infection	3 (3.1)	1 (0.9)	.34	2 (2.7)	0 (0)	1.00

IQR, interquartile range; LIMA, left internal mammary artery; NTSVG, no-touch saphenous vein graft; RAD, radial artery; RIMA, right internal mammary artery.

**Figure 1:**
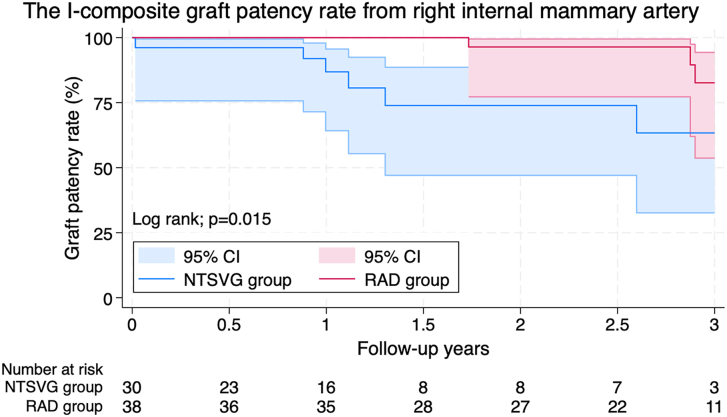
Kaplan-Meier curves show a significantly better coronary computed tomography angiography patency rate in the radial artery (RAD) group than in the no-touch saphenous vein graft (NTSVG) group (*P* = .015).

**Figure 2:**
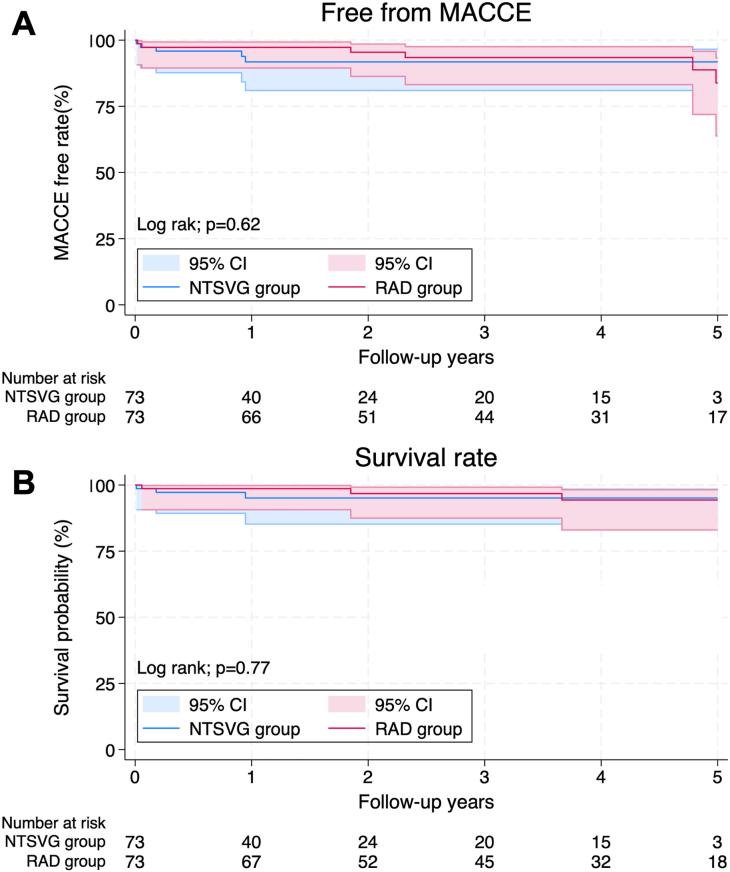
(a) Major adverse cardiac or cerebrovascular events (MACCE) free (*P* = .62) and (b) survival rate (*P* = .77) at 5 years after the operation. (NTSVG, no-touch saphenous vein graft; RAD, radial artery.)

Figure 1 demonstrates the differences in patency rates for the I-composite RIMA graft between the 2 groups. The coronary CTA patency rate was significantly better in the RAD group than in the NTSVG group (*P* = .015). The Kaplan-Meier curves showed no significant differences in freedom from MACCE (*P* = .62) and overall survival (*P* = .77) between the 2 groups (Figure 2).

## Comment

Bilateral IMA grafting is associated with superior long-term survival and lower MACCE rates compared with single IMA use,^2^ yet the optimal configuration for bilateral IMA continue to be debated. The RIMA is often used to revascularize the left coronary system, especially as a Y-composite from the LIMA or a V-composite from a SVG.^3^^,^^4^ Prior studies report better outcomes when the RIMA is used for the left coronary system than for the RCA, where patency tends to be lower.^5^ Nonetheless, in anaortic OPCAB, I-composite RIMA grafting to the RCA is often required to achieve complete revascularization. In our study, nearly all patients achieved complete revascularization with this configuration.

Sequential anastomosis using in-situ IMA alone may have limited patency, particularly when the distal portion with a smaller caliber is used.^6^ Extension grafts, such as the RAD and NTSVG, can overcome this limitation by offering greater length, diameter, and flexibility. In both unmatched and matched cohorts, ∼25% to 28% of patients underwent sequential anastomoses using these conduits, highlighting their utility. Previous work has shown superior long-term patency of I-composite IMA–RAD grafts compared with free RAD grafts.^7^

Although randomized trials have shown better patency and reduced MACCE with RAD grafts over conventional SVGs,^8^ the NTSVG technique is recognized for its potential to improve outcomes and may offer patency comparable to arterial grafts in selected cases. In this study, the lower patency observed in the NTSVG group could be due to caliber mismatch between the RIMA and thigh-harvested vein graft, potentially disturbing flow dynamics. Nevertheless, this did not translate into worse clinical outcomes, possibly because most I-composite grafts were deployed to the RCA, a territory less predictive of long-term survival.^9^

This study has several limitations. The overall sample size and the number of patients undergoing CTA were limited, and the follow-up period was relatively short. Although PS matching was applied, selection bias remained because the NTSVG group tended to include higher-risk patients. The standardized mean difference for the EuroSCORE II exceeded the acceptable threshold (−0.15), indicating residual imbalance. Furthermore, unmeasured confounders may have affected the results. Future studies with larger cohorts and longer follow-up periods are warranted.

In conclusion, although the RAD and NTSVG can both be safely used as extension grafts in I-composite RIMA configurations during anaortic OPCAB, the RAD demonstrated superior midterm patency. These findings support the RAD as a practical and durable conduit choice for multivessel anaortic OPCAB.

## Figures and Tables

**Supplementary Figure:**